# Seasonal environmental factors drive microbial community succession and flavor quality during acetic acid fermentation of Zhenjiang aromatic vinegar

**DOI:** 10.3389/fmicb.2024.1442604

**Published:** 2024-08-07

**Authors:** Xiaoting Ye, Yongjian Yu, Jiaxin Liu, Yuanyuan Zhu, Zhen Yu, Peng Liu, Yuqin Wang, Ke Wang

**Affiliations:** ^1^School of Grain Science and Technology, Jiangsu University of Science and Technology, Zhenjiang, China; ^2^Jiangsu Provincial Engineering Research Center of Grain Bioprocessing, Zhenjiang, China

**Keywords:** ZAV, different seasons, differential core microorganisms, environmental factors, flavor substances

## Abstract

This study investigated the impact of seasonal environmental factors on microorganisms and flavor compounds during acetic acid fermentation (AAF) of Zhenjiang aromatic vinegar (ZAV). Environmental factors were monitored throughout the fermentation process, which spanned multiple seasons. Methods such as headspace solid phase microextraction gas chromatography-mass spectrometry (HS-SPME-GC-MS), high performance liquid chromatography (HPLC), and high-throughput sequencing were employed to examine how these environmental factors influenced the flavor profile and microbial community of ZAV. The findings suggested that ZAV brewed in autumn had the strongest flavor and sweetness. The key microorganisms responsible for the flavor of ZAV included *Lactobacillus acetotolerans*, *Lactobacillus plantarum*, *Lactobacillus reuteri*, *Lactobacillus fermentum*, *Acetobacter pasteurianus*. Moreover, correlation analysis showed that room temperature had a significant impact on the composition of the microbial community, along with other key seasonal environmental factors like total acid, pH, reducing sugar, and humidity. These results provide a theoretical foundation for regulating core microorganisms and environmental factors during fermentation, enhancing ZAV quality.

## 1 Introduction

Zhenjiang aromatic vinegar (ZAV) is one of the four renowned vinegars in China. Traditional AAF processing typically employs an open, multistrain combined fermentation technique (Huang et al., 2022). Within this process, intricate microbe metabolic actions are instrumental in contributing to superior vinegar flavor quality (Li et al., 2023). Notably, compared to controlled single-strain liquid fermentation, open multi-strain fermentation yields a more spectrum flavored vinegar, albeit these flavors being more vulnerable to external influences, leading to notable variation in quality. Through years of refinement and genetic infusion, ZAV’s brewing environment has resulted in a distinct microbial ecosystem and the exclusive ZAV flavor (Liu et al., 2023). Presently, substantial investigation surrounds the influence of environmental variables on the microbial community structure and flavor compound profile of traditional ZAV. Huang et al. (2022) utilized molecular ecology to scrutinize shifts in the cognate microbes, environmental elements, and flavor metabolites present throughout ZAV’s AAF phase, thus providing a scientific rationale behind the role played by the acetate fermentation process in flavor metabolite formation.

Environmental variables, including air, water, and soil microbiota, considerably shape the fermentation microbiome. Seasonal shifts modify these elements, thereby influencing flavor evolution in fermented goods (Zhou et al., 2020; Fang et al., 2021; Ma et al., 2022). During open multi-strain solid-state fermentation, varying climactic conditions along seasonal gradients influence the microbial consortium, which impacts their metabolic capacity, subsequently instigating flavor quality disruption in fermented edibles. Although substantial research exists regarding environmental effect on the bacterial community structure and flavor profile of traditional ZAV, seasonal aspects remain understudied (Huang et al., 2021; Zhou et al., 2021). Seasonal transitions markedly impact the bacterial makeup, aroma, and quality of fermented food items. Elements like pH, ethanol, reducing sugar, and titratable acidity guide the microbial succession during fermentation, leading to fluctuating species redundancy at distinct production stages (Yu et al., 2012; Liu et al., 2023). To bridge these comprehension voids, we intend to thoroughly comprehend the environmental impact on central brewing microbes and flavor compound biosynthesis in ZAV across distinct seasonal zones. Through a yearly viewpoint, we aim to gain a holistic insight into the microbial community progression and flavor synthesis. This research paves the way towards comprehending the traditional brewing mechanisms and deploying precision quality management in vinegar manufacturing.

In this investigation, we observed seasonal fluctuations of environmental variables during ZAV bioconversion, delineating the altering dynamics of microbial communities and flavor constituents. With O2PLS analysis, we pinpointed key microbes in relation to flavor components across these seasons. Furthermore, spearman correlation and RDA evaluations revealed microbiota correlates with environmental factors across all four seasons. Overall, this research will contribute to a deeper understanding of the influence of environmental factors on ZAV fermentation, paving the way for improved control of flavor quality and promoting the modernization of solid-state vinegar fermentation (see Table 1).

**Table 1:** Taste analysis of free amino acids in vinegar fermented grains after acetic acid fermentation in different seasons.

Amino acid	Threshold (mg/100 g)	TAV			
		SP18	SU18	AU18	W18
Asn	100.00	2.81 ± 0.64	1.72 ± 5.48	4.65 ± 54.44	3.15 ± 13.89
Gln	30.00	14.74 ± 0.42	1.12 ± 0.15	29.48 ± 3.58	7.18 ± 1.55
Ala	60.00	22.94 ± 0.4	2.92 ± 0.9	29.77 ± 3.24	23.08 ± 4.27
Gly	130.00	2.62 ± 1.53	0.92 ± 1.16	3.8 ± 0.12	2.74 ± 1.56
Ser	150.00	3.37 ± 1.17	1.26 ± 10.15	4.42 ± 2.83	3.16 ± 2.27
Thr	260.00	1.29 ± 0.4	0.51 ± 5.67	1.63 ± 3.02	1.27 ± 1.24
Arg	50.00	17.46 ± 0.77	9.11 ± 12.59	15.61 ± 0.61	32.65 ± 2.58
His	20.00	11.3 ± 0.54	25.8 ± 4.14	8.78 ± 2.77	11.76 ± 17.5
Ile	90.00	4.39 ± 12.15	4.73 ± 2.96	6.04 ± 0.34	4.65 ± 2.79
Leu	190.00	5.17 ± 1.58	0.51 ± 13.9	5.61 ± 4.25	5.14 ± 5.24
Lys	50.00	8.21 ± 4.92	4.92 ± 10.33	11.16 ± 0.9	8.69 ± 2.08
Met	30.00	7.12 ± 0.2	7.18 ± 3.41	8.51 ± 2.27	6.11 ± 2.62
Phe	90.00	6.62 ± 3.83	0.16 ± 16.49	6.07 ± 3.85	6.94 ± 1.8
Val	40.00	18.34 ± 1.69	0.32 ± 0.8	21.22 ± 1.28	16.89 ± 1.72
Tyr	ND	—	—	—	—

(ND: not found, so the TAV value cannot be calculated; TAV value: indicates the ratio of the content of each odorant to the odorant threshold of each amino acid in water. When the TAV value is less than 1, it is considered that the substance has no contribution to the presentation of taste, and when the TAV value is greater than 1, it is considered that the substance has a contribution to the presentation of taste).

## 2 Materials and methods

### 2.1 Reagents

The DNA extraction kit was purchased from Magen Biotechnology Co., Ltd. (Guangzhou, China). Other commercial chemicals used in this study are analytically pure and purchased from Sinopharm Chemical Reagents Co., Ltd. (China).

### 2.2 Preparation of samples

**Figure 1:**
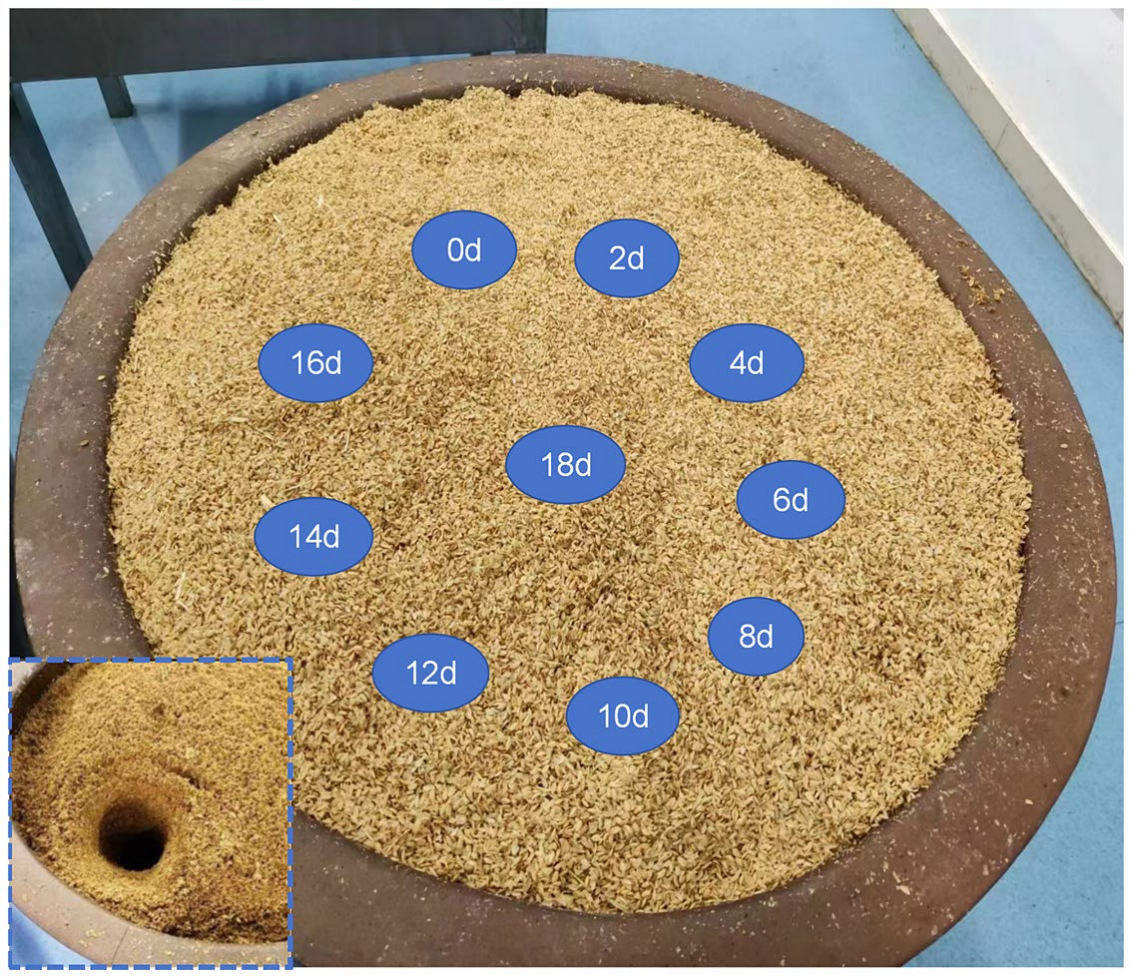
Sampling diagram of vinegar *pei* during AAF process using ZAV.

ZAV was produced through a traditional solid-state fermentation process. The experimental samples for this study were artificially cultivated vinegar *pei* from four different seasons of ZAV: spring (SP) in March, summer (SU) in June, autumn (AU) in September, and winter (W) in December. The Vinegar *pei*, a microorganism-rich solid–liquid blend derived from AAF, served as experimental specimens and were collected on the 0 day, 2 days, 4 days, 6 days, 8 days, 10 days, 12 days, 14 days, 16 days, and 18 days of fermentation, with each sample collected three times. Vinegar was extracted from the fermenter in a top-to-bottom manner, and 100 g of it was weighed and uniformly mixed before being transferred to a self-sealing bag (Figure 1). A 30 mL sample of the marinade was obtained from the bottom of the cylinder and stored at −20°C in a 50 mL centrifuge tube. Before analysis, the samples were thawed in a water bath at room temperature (26°C to 30°C).

### 2.3 Monitoring of environmental factors

This research scrutinized the inner and outer environmental conditions during ZAV fermentation, including the vinegar *pei*’s acid content, pH level, sugars, organic acids, amino acids, and temperature; and, the surrounding environment dimensions: temperature and relative humidity. The ground herbs fermented with vinegar were blended with 30 mL of water, then stirred for 3 h at 100 rpm and 20°C in a well-controlled incubator. pH, total acidity, reducing sugars, yeast alcohol respectively, were quantified using the methods suggested by Yu et al. (2012), Huang et al. (2022), and Liu et al. (2023). Temperature data was reported with an accurate thermometer, while humidity levels were calculated through a hygrometer from Shanghai Kuangjian Instrument Technology Co., Ltd. And Zhejiang Ningbo Deli Tool Co., Ltd., respectively. These experiments were completed three times. Subsequently, the concentration of organic acids was evaluated via HPLC analysis with a Waters e2695 equipment and a 2,998 PDA detector from Yu’s et al. (2012) prior study. Lastly, the amounts of volatile molecules were confirmed with an HS-SPME/-GC/MS system mentioned by Huang et al. (2022).

### 2.4 DNA extraction, identification, and sequencing

Genomic DNA was extracted using HiPure Universal DNA Kit (D3018, Magen, Guangzhou), following the manufacturer’s instructions. The V3–V4 region of the 16S rRNA gene was amplified using universal primers 338F and 806R, according to the method of Wang et al. (2016), while the ITS region was amplified using primers ITS1F and ITS2. The samples were double-ended sequenced on an Illumina MiSeq platform (Illumina, San Diego, California, United States). All measurements were performed in triplicate.

### 2.5 Statistical analysis

Preliminary statistical analysis of the data was conducted in Microsoft Office’s EXCEL software (2016 version). The Chiplot platform’s bubble chart analysis was utilized to examine the content changes of organic and amino acids during acetic acid fermentation. To further explore seasonal differences in organic acid fermentation, Chiplot’s grouping error histogram was used. The Chiplot website can be accessed at https://www.chiplot.online. The Vigan package (version 3.6.3, R Core Team) in R was employed to carry out redundancy analysis (RDA). The OmicStudio tool (https://www.omicstudio.cn, Lianchuan Biotechnology Co., Ltd.) clustered the signed correlation heat map. R’s VennDiagram package (version 3.6.3, R Core Team) was used to generate the VennDiagram. SIMCA (14.1 edition, Sartorius, Göttingen) calculated O2PLS, with flavor substances synthesized during acetic acid fermentation as the independent variable (*X*) and microbial flora in vinegar fermented grains as the independent variable (*Y*), and fitted by O2PLS-DA model. Significant (*p* < 0.05) high correlation (|*ρ*| > 0.7) was visualized using the R package igraph (version 3.6.3, R Core Team). Lastly, GraphPad Prism (version 8.0.1, GraphPad Software) and Cytoscape software (version 3.5.1, Free Software Foundation) were utilized.

## 3 Results

### 3.1 Monitoring of internal and external environmental factors of ZAV in different seasons

Given open fermentation practices in ZAV brewing, varying ambient conditions could significantly influence the microfloral ecosystem within vinegar fermented grains, causing volatile aromatic variations during acetic acid fermentation. Therefore, to comprehend the monsoons’ influence on ZAV production, we examined variables such as room temperature, humidity, grain temperature, total acidity, pH, and glucose levels at dissimilar seasons. A correlation analysis also evaluated the impact these environmental conditions had on ZAV’s key diverse microorganisms.

**Figure 2:**
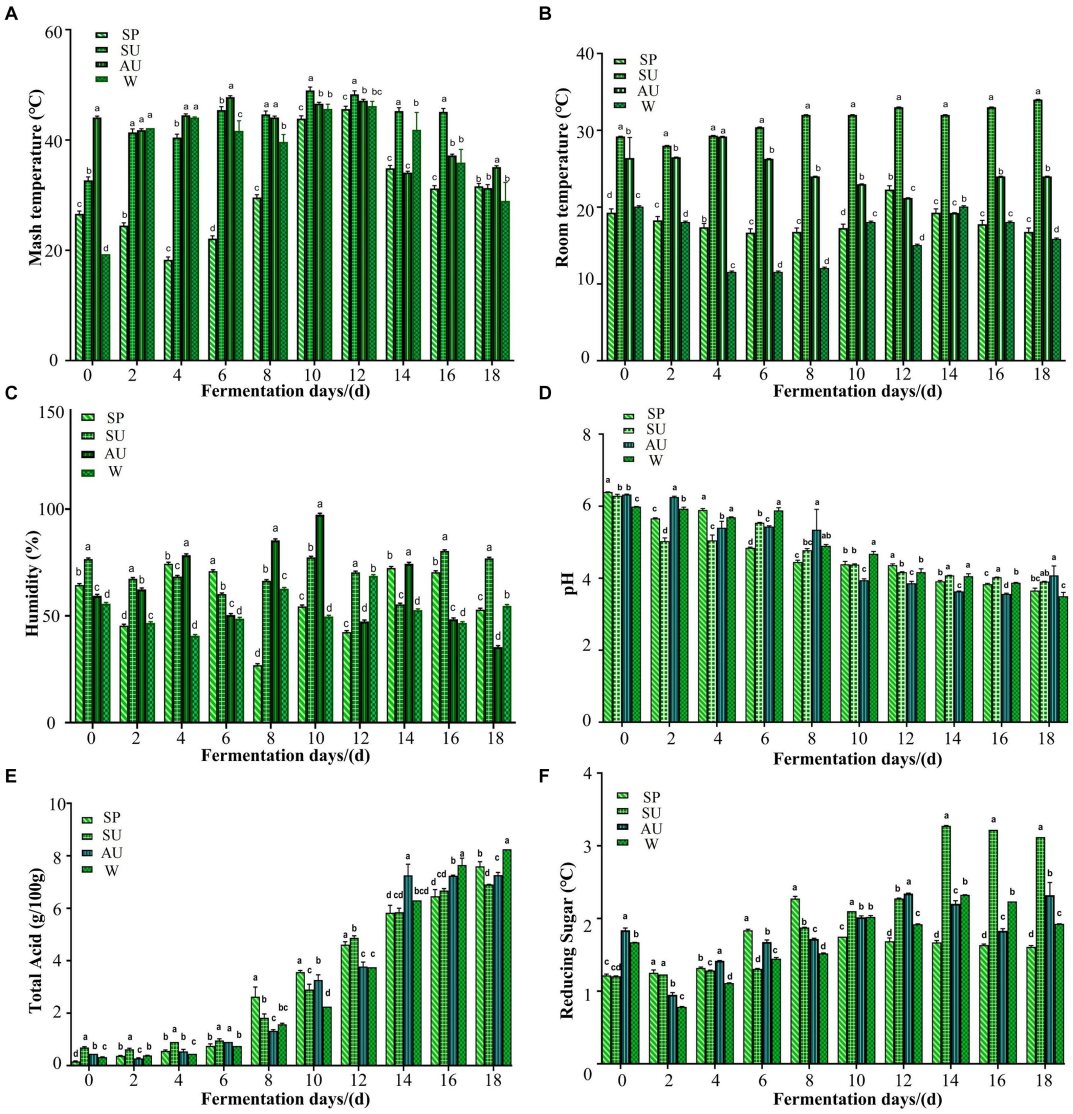
Analysis on the differences in changes of internal and external environmental factors in vinegar fermented grains of ZAV in different seasons. **(A)** Analysis on the difference of temperature changes inside the vinegar *pei* during acetic acid fermentation of ZAV in different seasons. **(B)** Analysis on the difference of temperature changes outside the vinegar *pei* during acetic acid fermentation of ZAV in different seasons. **(C)** Changes in the humidity of vinegar *pei* during the fermentation process of Zhenjiang fragrant vinegar. **(D)** Changes in pH during fermentation of ZAV. **(E)** Changes in total acid during fermentation of ZAV. **(F)** Changes in reducing sugar during the fermentation of ZAV.

During seasonal variations, marked fluctuations were observed in the internal/external temperature dichotomy of brewed grains (Figures 2A,B). Internal heat variations correlate with metabolic activities. Acetic fermentation revealed a gradual rise in grain temperatures, though they subsequently leveled off at ambient levels. Autumn exhibited peak grain temp, trailed by summer, 8 days pre-fermentation commencements. Post day 10–day 12, seasonal medians stood at 49°C (SU), 47°C (AU), 46°C (W), and 45.6°C (SP). The late phase saw organisms initiate stable growth phases and grain temps decline to approximately 37°C post acetic fermentation halt. Environmental humidity is crucial for vinegar flavor intensity (Agma Okur et al., 2022). Figure 2C illustrates considerable humidity variances during various seasons’ acetic fermentations, where spring/winter registers the lowest humidity versus spring/autumn. Humidity peaks as high as 77% (SU) and 97% (AU) in 8 days and 10 days, respectively. This phenomenon is elucidated through linking internal/external temperature shifts during the brewing process. Microbial metabolism and breeding create biothermal energy, reduce the moisture content in grains.

The total acidity and pH levels serve as critical metrics assessing vinegar strength and unequivocally influence product quality (Sedjoah et al., 2021). Figure 2D illustrates a precipitous drop in pH from day 0–day 8, followed by minimal fluctuations from 2 days–4 days, attributed to microbial activity metabolizing nutrient sources into organic acids beyond acetic and lactic acids, thereby imparting ZAV’s mild flavor profile. Figure 2E documents a steady rise in total acid throughout the fermentation process. The total acid content changes almost uniformly in each season, showing a gradually increasing trend as fermentation progresses. It is worth noting that the total acidity in summer and spring is higher than that in autumn and winter. Winter displays the maximum amount of total acid accumulation (8.3 g/100 g), with summer showing the least (6.9 g/100 g).

Reducing sugar is a crucial physical and chemical indicator used to measure the flavor quality of vinegar (Mat Isham et al., 2019). During summer, the reducing sugar content is higher than in the other three seasons, gradually increasing from 1.20 g/100 g of fermented grains on fermentation 0 day to a maximum of 3.27 g/100 g of fermented grains on fermentation day 14. In autumn and winter, the content of reducing sugar decreases from 0 day to 2 days due to more favorable microbial growth and reproduction conditions, leading to increased acid production of microbial metabolism as fermentation progresses (Figure 2F). Yeast and lactic acid bacteria use the reducing sugar produced by previous microbial metabolism in vinegar fermented grains to produce alcohol and lactic acid. Additionally, some reducing sugar participates in the Maillard reaction, leading to a gradual decrease in the reducing sugar content. At the end of acetic acid fermentation, the content of reducing sugar is highest during summer (3.12 g/100 g dry fermented grains).

### 3.2 Analysis on the structural changes of flavor substances in ZAV during different seasons

**Figure 3:**
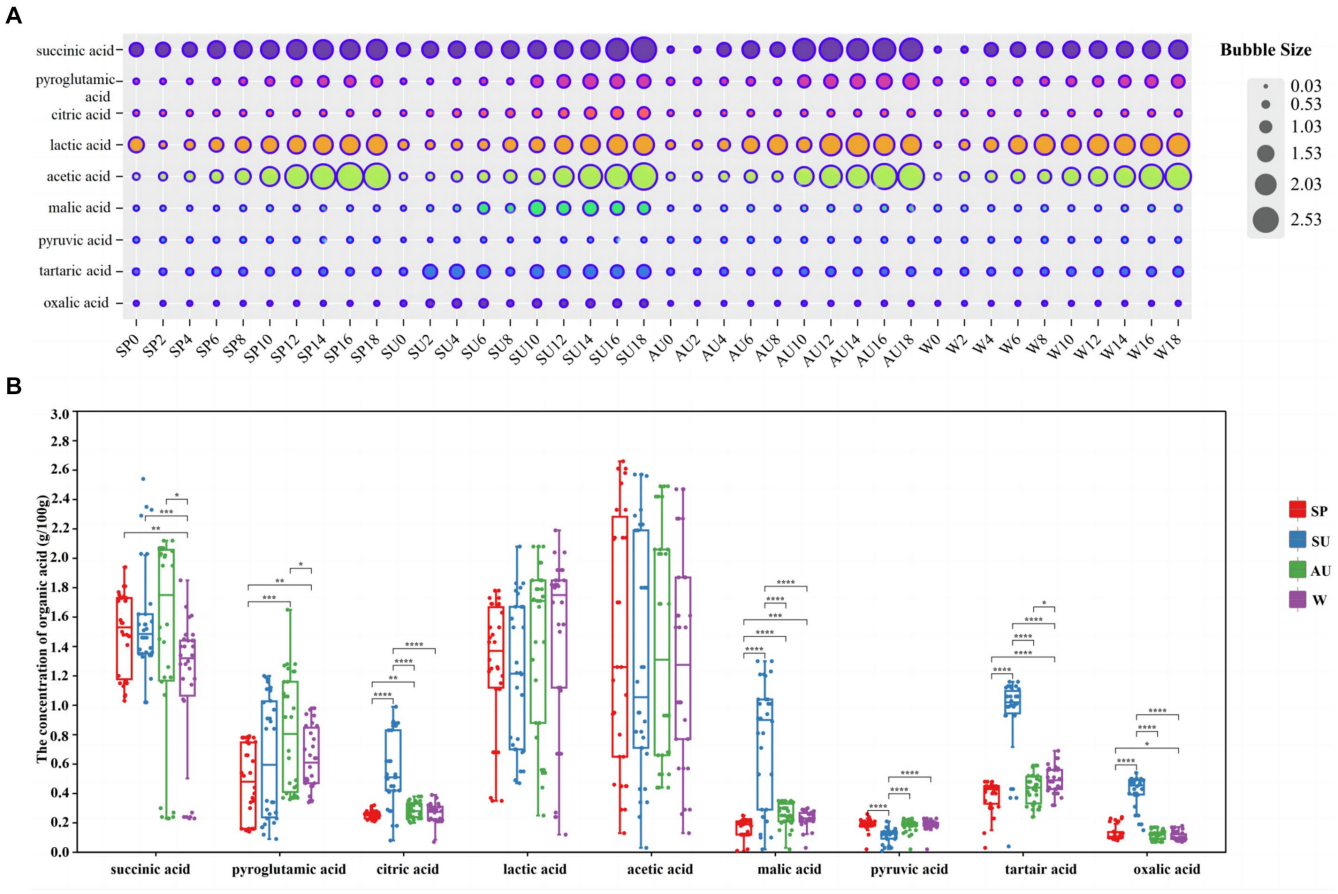
Analysis of changes in organic acid content in vinegar *pei* during solid-state fermentation of ZAV in different seasons. **(A)** Analysis of the content changes of nine organic acids during acetic acid fermentation in different seasons bubble chart. **(B)** Seasonal difference analysis of nine organic acids.

ZAV, abundant in various organic acids such as acetic acid, imparts a subtle sourness devoid of astringency, distinguishing it from other vinegars (Yu et al., 2012). The concentration of these acids markedly affects the vinegar’s fragrance and palatability (Noh et al., 2020). Using HPLC, we examined fluctuations in nine distinct organic acids during AAF in ZAV. Lactic and acetic acids emerged as key components. With fermentation progression, the concentration of all nine increased, indicating noticeable seasonal variations (Figures 3A,B). Post-fermentation, acetic acid exhibited peak levels in spring, lactic acid in winter, succinic acid in summer, pyroglutamic acid in autumn, tartaric, citric, and oxalic acids in summer, differing significantly for the remaining seasons.

**Figure 4:**
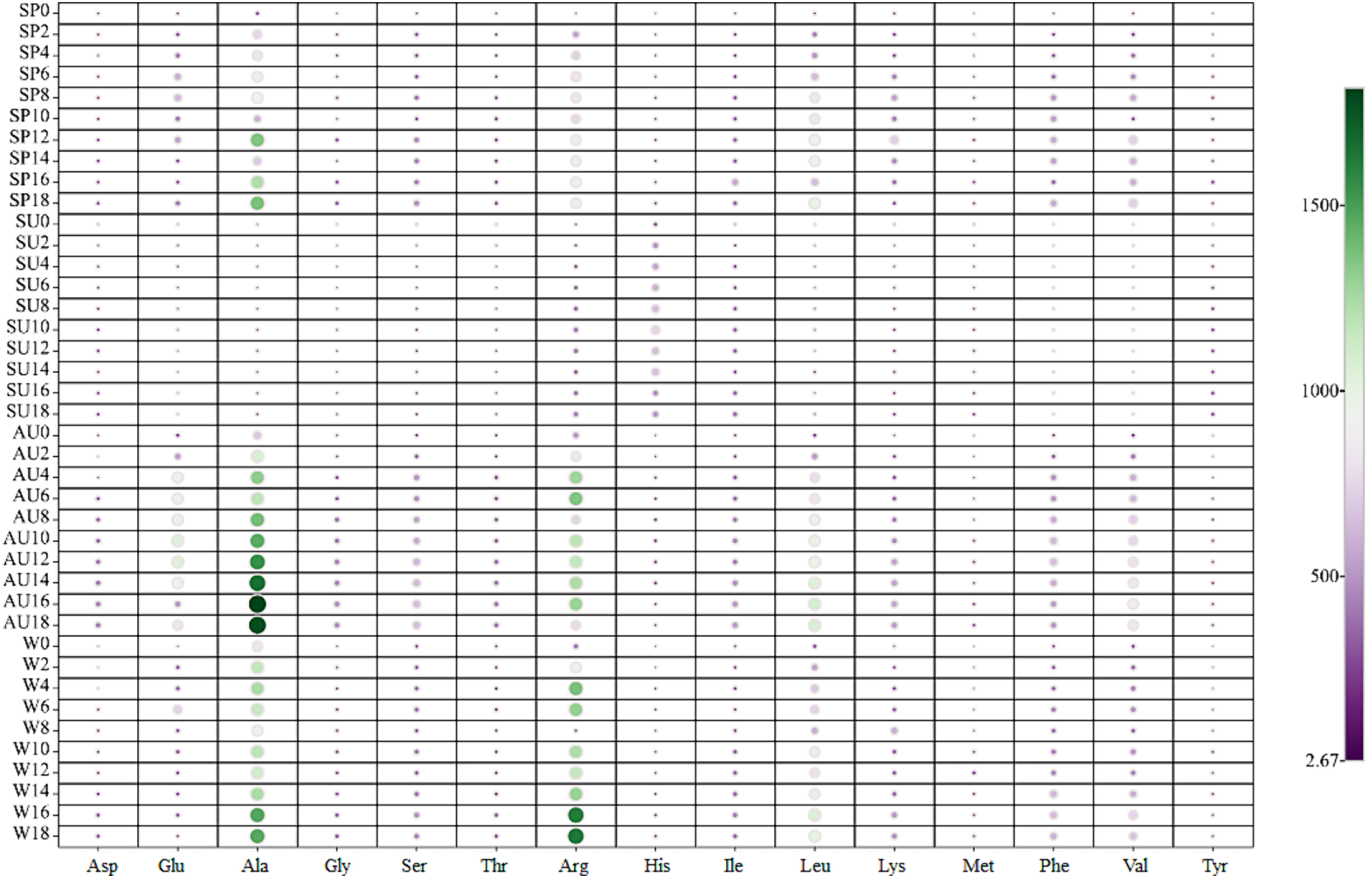
Differences in the composition of free amino acids in ZAV during different seasons.

Amino acids, key flavoring agents in vinegar, regulate its taste (Zhang et al., 2019). Free amino acids were analyzed in the fermentation process of ZAV across four seasons using an automatic amino acid analyzer (Figure 4 and Supplementary Table S1). Results demonstrate an initial increase followed by a decrease in the content of amino acids in all 15 types of vinegar, with the highest levels measured in autumn. As fermentation proceeds, alanine and arginine increase in fermented vinegar, exceeding other free amino acids. At the end of fermentation, the highest total content of alanine was in autumn and arginine in winter. TAV describes the ratio of each odorous substance content to the odor threshold of each amino acid in water (Gao et al., 2022). We calculated the TAV values of 16 odorous amino acids in ZAV at the end of fermentation to assess quality differences across seasons. Our findings highlight the most significant variation as between ZAV in summer and the other seasons. The TAV values of aspartic acid and glutamic acid, which smell fresh in autumn, and sweet amino acids such as alanine, glycine, serine, and threonine, have the highest values. Meanwhile, bitter-tasting amino acids have the highest values in winter. Overall, ZAV has a pronounced umami flavor and sweetness in autumn, with the bitter taste most prominent in winter.

**Figure 5:**
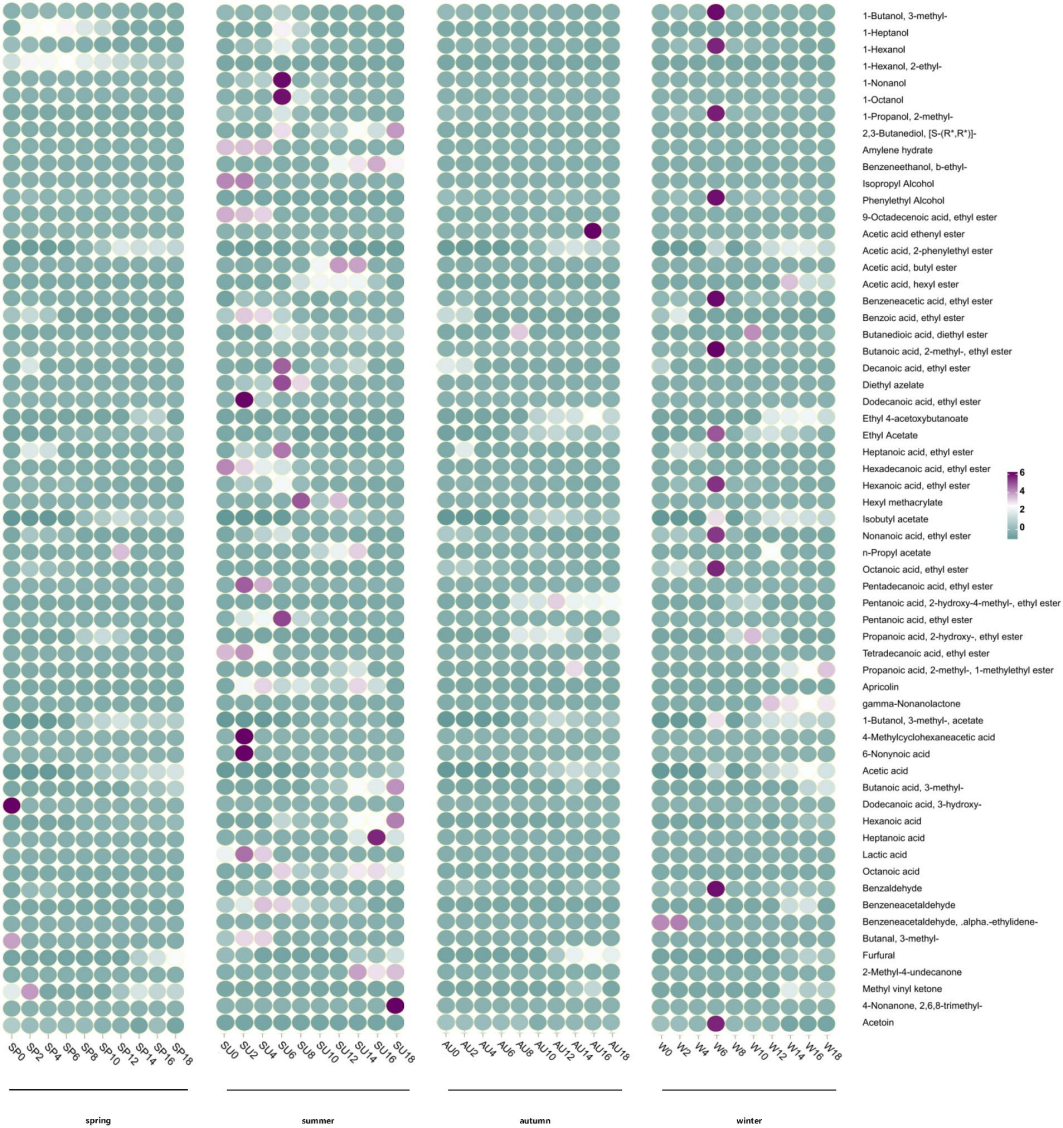
Dynamic changes of volatile flavor substances in vinegar fermented grains during acetic acid fermentation in different seasons.

The volatile flavor substances, both in terms of their types and contents, are significant indicators for evaluating vinegar quality (Fang et al., 2021). Volatile compounds present in vinegar impart a distinct flavor but are relatively free compared to organic acids. The concentration of amino acids is low; however, their presence is crucial in enhancing the harmonious and rich taste of vinegar (Charles et al., 2000). In this study, GC–MS was used to detect the volatile substances present in ZAV acetic acid fermentation during different seasons, and to analyze the dynamic changes in volatile substances and flavor compounds resulting from these seasonal variations (Figure 5). Our findings revealed a total of 62 flavor compounds, consisting of 12 alcohols, 9 acids, 31 esters, 5 aldehydes, and 4 ketones. Twelve alcohols, including 3-methyl-1-butanol, isopropanol, and phenylethanol, have been identified in AAF. These alcohols primarily act as synthetic precursors during the fermentation process, leading to the production of other volatile flavor compounds in microbial metabolism (Xu et al., 2022). The total content of volatile alcohols was highest at the end of the fermentation cycle during autumn. Esters are the most significant contributors to the unique aroma of vinegar (Huang et al., 2022). In the entire process of ZAV acetic acid fermentation, the highest concentration of volatile esters, such as ethyl acetate and ethyl lactate, is directly related to their synthetic precursors, acetic acid, and lactic acid. After that, increase (Wang et al., 2021). The highest content of volatile esters was observed during summer. Acetaldehyde, furfural, and phenylacetaldehyde are the primary aldehydes present in vinegar that provide a floral fragrance. Notably, significant differences in the volatile flavor compounds were observed across the different seasons, with the richest total content of volatile flavor compounds occurring during autumn. By the end of the fermentation process, the highest total content of volatile alcohols and ketones was noted during autumn, while the highest total content of volatile esters and acids was observed during summer. Finally, the highest total content of volatile aldehydes was found during winter. Among all seasons, autumn has the most significant concentration of volatile aldehydes. Acetoin, which imparts a cream yogurt-like aroma, and 2,3-butanedione, responsible for the buttery aroma, are the main ketones present in fermented grains. These two ketones, along with 2,3-butanediol, are precursors to functional flavor compounds such as ligustrazine, which imparts a nutty and baked aroma (Zhou et al., 2017). The highest concentration of volatile ketones was observed during autumn at the end of the fermentation period.

### 3.3 Analysis on the changes of microbial colony structure in ZAV during different seasons

**Figure 6:**
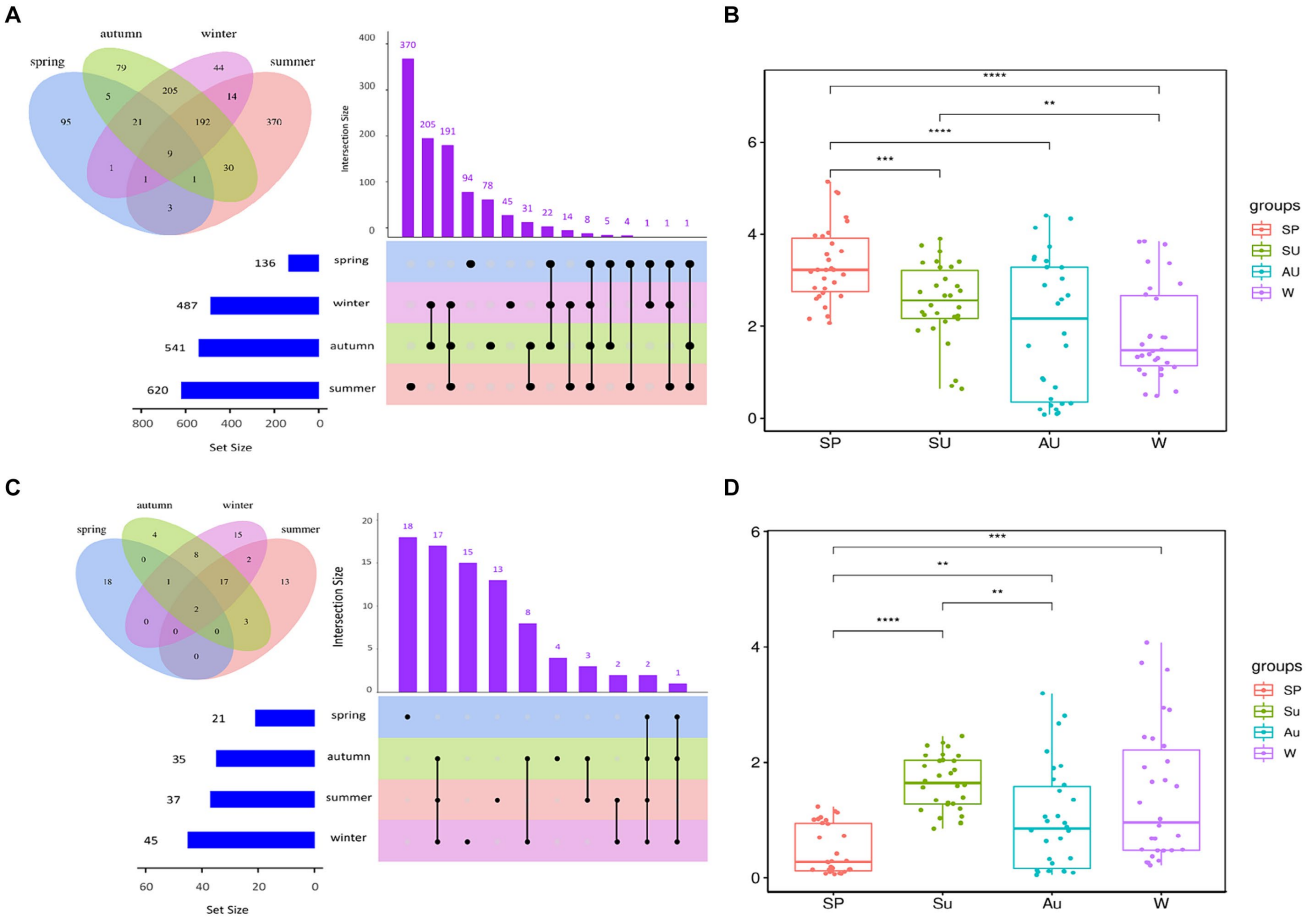
Analysis of bacterial diversity in ZAV vinegar *pei* in different seasons. **(A)** Upset diagram of bacteria and microorganisms in different seasons. **(B)** Box plot of differences in bacterial Shannon index between groups in different seasons. **(C)** Upset diagram of fungi and microorganisms in different seasons. **(D)** Box plot of differences in fungi Shannon index between groups in different seasons.

**Figure 7:**
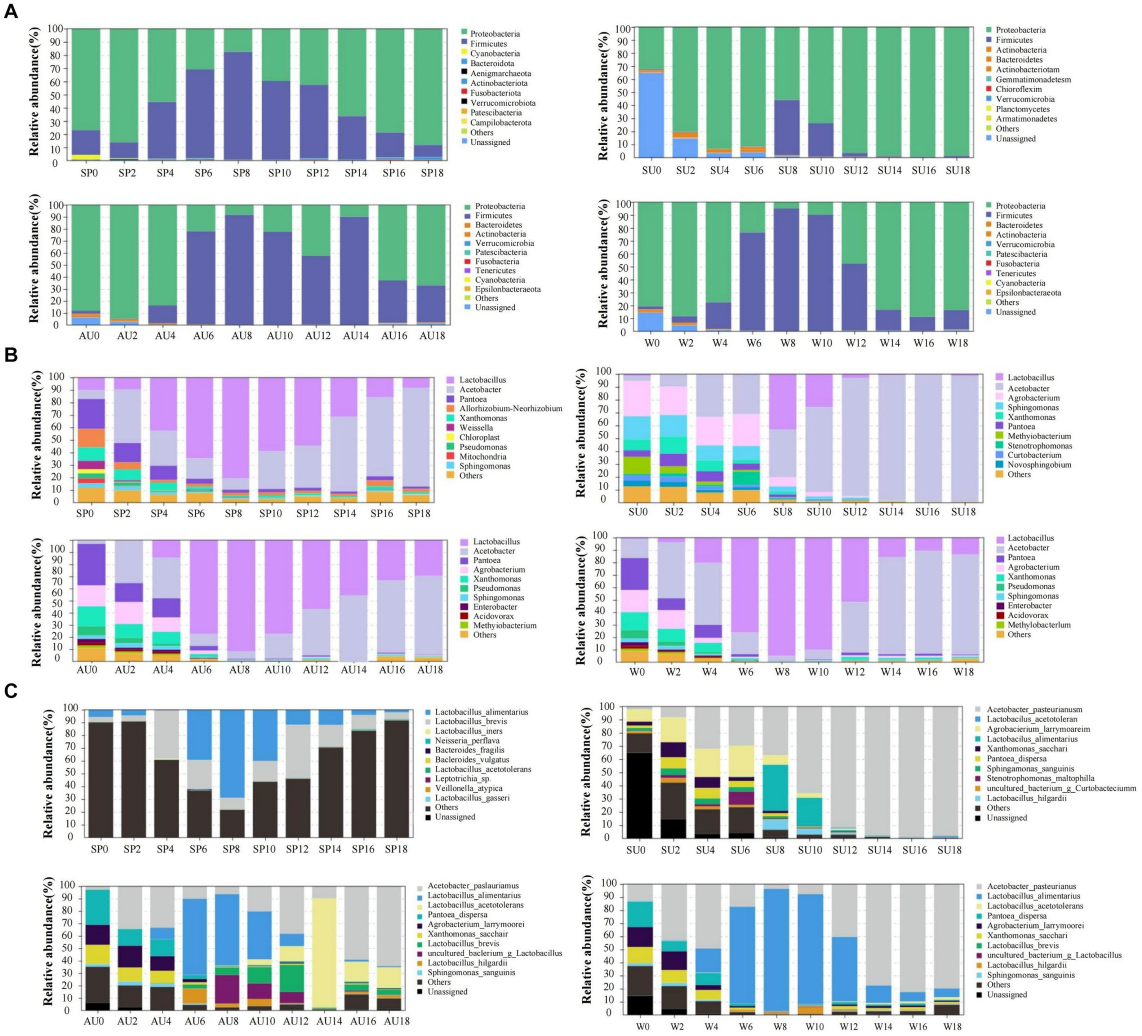
Analysis of changes in bacterial composition and structure in ZAV vinegar *pei* during different seasons. **(A)** Phylum level. **(B)** Genus level. **(C)** Species level.

**Figure 8:**
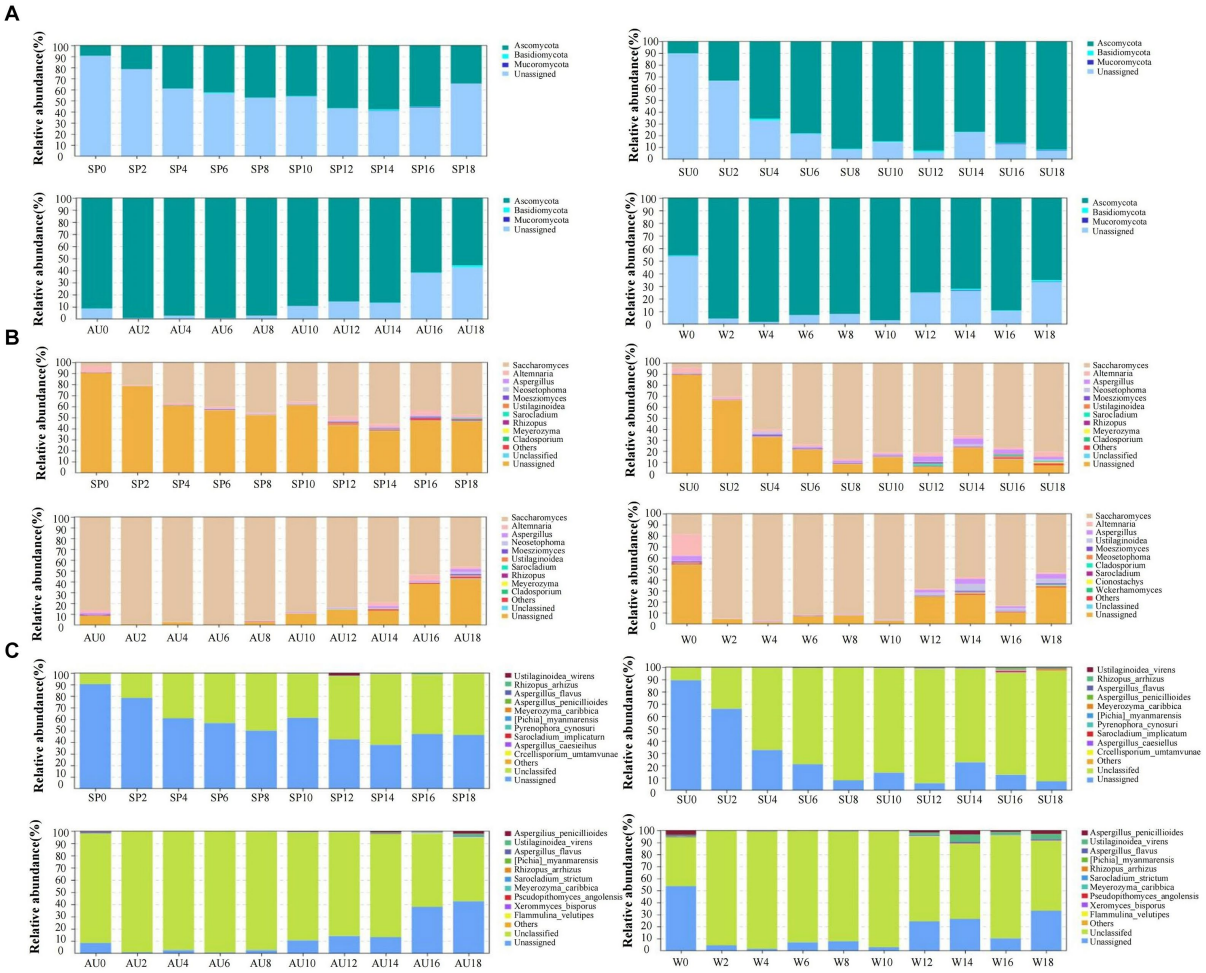
Analysis of changes in fungi composition and structure in ZAV vinegar *pei* during different seasons. **(A)** Phylum level. **(B)** Genus level. **(C)** Species level.

The rapid changes in microbial community composition structure and abundance during AAF were closely linked to ZAV’s unique “layer-by-layer fermentation” process (Wang et al., 2016). The diversity and structural changes of the ZAV microbial community in different seasons were analyzed using the high-throughput sequencing technique (Figure 5). The results indicated that the microbial community varied significantly across different seasons. In summer, the number of bacteria increased due to the high temperature and humidity, resulting in an increased diversity of microbial species in the vinegar fermented grains, including 620 types of bacteria. The fungi were most abundant in winter (Figures 6A,C). Furthermore, the microbial community differed significantly between spring and the other seasons and the fungal community differed significantly between summer and autumn (Figures 6B,D). The diversity of the microbial community at the phylum level during vinegar grain fermentation was analyzed and compared across seasons by comparing the data of highly abundant microbial communities. The results indicated that Firmicutes and Proteus dominated the bacterial microflora in ZAV vinegar throughout all seasons. The fungal flora in vinegar fermented grains belonged to four phyla: Ascomycetes, Basidiomycetes, Mucor, and Unassigned. Among them, Ascomycetes dominated the fermentation process, and its relative abundance was the highest in autumn (Figure 7A and Figure 8A).

*Acetobacter* and *Lactic acid bacteria* are the dominant bacterial genera in fermented grains (Top 10 relative abundance of OTUs). The highest relative abundance of *Acetobacter* is observed in summer, while that of *Lactobacillus* is low in the middle of the four seasons. The highest relative abundance of *Panthera* is observed in autumn, *Agrobacterium* in summer, *Xanthomonas* in autumn, and *Sphingomonas* in summer. In addition, *Saccharomyces*, *Alternaria*, *Aspergillus*, and *Uassigned* are the predominant fungal microorganisms, with *Saccharomyces* showing the highest relative abundance in autumn, *Alternaria* in winter, and *Aspergillus* in summer (Figures 7B, 8B). The selective pressure of acidic environment in acetic acid brewing inhibits the growth of bacteria sensitive to acidic environments. At the same time, the heat generated by microbial fermentation (with peak temperatures ranging between 45–48°C) can kill heat-labile bacteria, thereby stabilizing the community structure in fermented grains under environmental pressure. Oxygen molecules inside the vinegar grains during AAF are rapidly depleted in summer, and this promotes the growth and reproduction of anaerobic and facultative aerobic microbial flora. In summer, the relative abundance of *Acetobacter* and *Sphingomonas* was the highest across all seasons. *Xanthomonas* and *Pantoea*, on the other hand, had the highest abundance in autumn. For fungi, *Saccharomyces*, *Alternaria*, *Aspergillus*, and Unassigned were the dominant fungal microorganisms during AAF, consistent with existing research findings (Xu et al., 2011; Li et al., 2016). Yeast mainly comes from the early stages of wine fermentation, rapidly fermenting sugar to produce ethanol and polypeptides and providing nutrients for the growth and metabolism of other microbial communities in later stages (Cai et al., 2018). *Aspergillus* mainly decomposes macromolecular substances such as protein and starch by secreting enzymes like amylase and protease, providing small molecular nutrients like peptides and amino acids for other microbial communities in vinegar fermented grains (Meena et al., 2021). *Alternaria* is a common saprophytic and plant pathogenic bacterium in soil and air, with those in fermentation environments mainly coming from raw materials of fermented grains. As pH values decrease during fermentation, the number of Alternaria steadily declines. The relative abundance of *Saccharomyces* usually peaks during autumn, consistent with its preferred range of growth temperatures, which is between 20–30°C. In winter, the relative abundance of *Alternaria* is the highest, while that of *Aspergillus* is highest in summer.

Changes in bacterial strains are primarily reflected in fluctuations of *Lactobacillus foodborne*, *Lactobacillus acidophilus*, and *Acetobacter pasteurelloides*. Eight days before fermentation, the abundance of *Lactobacillus foodborne* and *Acetobacter pasteurelloides* have the biggest influence, while common changes of *Lactobacillus foodborne*, *Lactobacillus acidophilus*, and *Acetobacter pasteurelloides* dominate the later stages of fermentation. Throughout the fermentation process, numerous low-abundance fungal species are present in the fermented grains (Figures 7C, 8C).

### 3.4 Correlation analysis of flavor substances and differential core microorganisms in different seasons of ZAV

**Figure 9:**
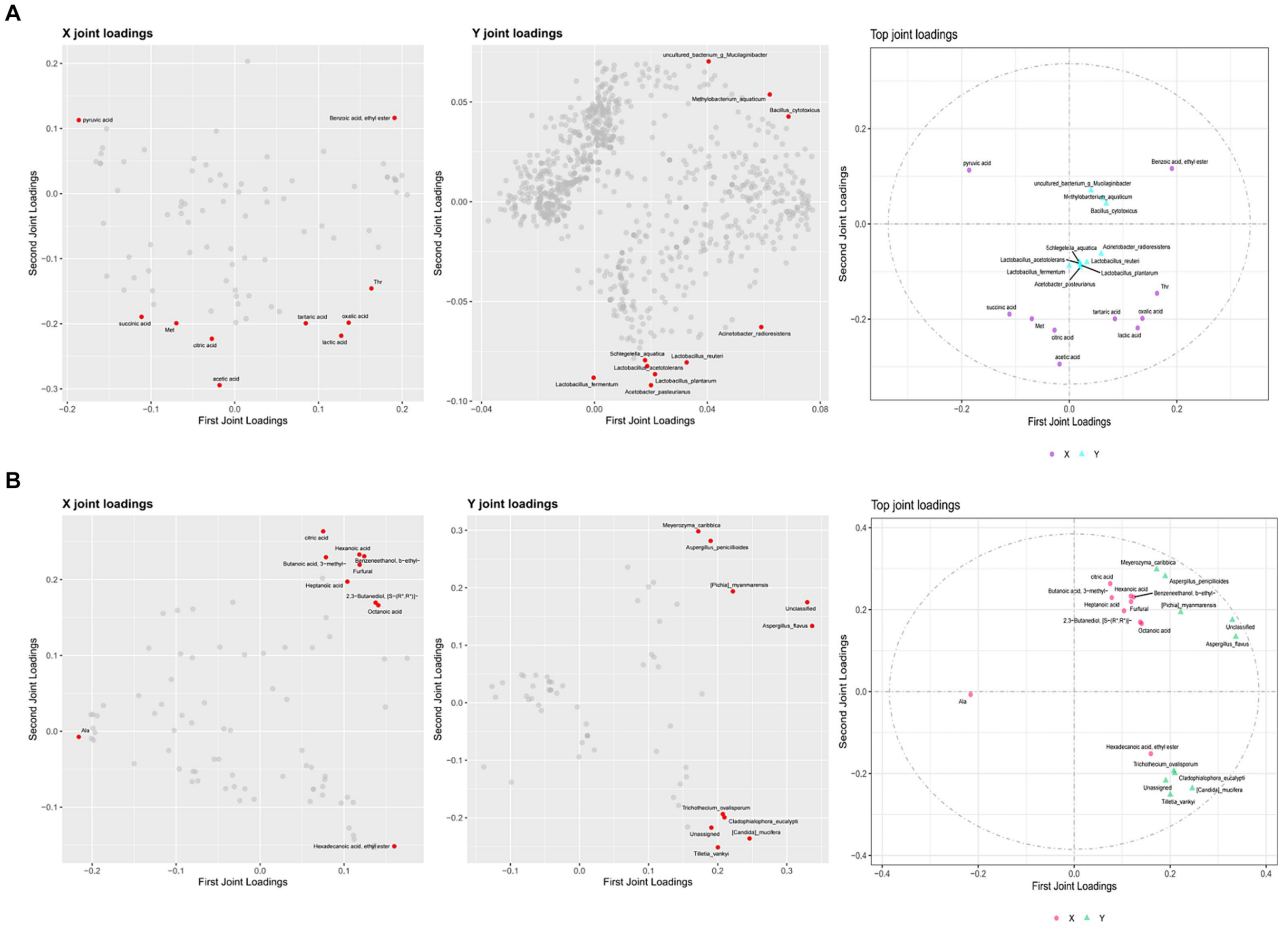
O2PLS load diagram and associated load diagram of flavor substances and microorganisms of ZAV in different seasons (top 10). **(A)** Bacterium. **(B)** Fungus.

To identify the correlation between flavor compounds and microorganisms in different seasons of ZAV, this study generated various omics loading plots for relevant variables (such as flavor compounds or microorganisms in different seasons). The loading values were analyzed, and the top 10 flavor compounds and microorganisms with the highest square sum of the two dimensions were identified and integrated into the loading plots. Figure 9 and Supplementary Table S2 display the highly correlated flavor compounds and microorganisms, including *Acetobacter pasteurianus*, *Lactobacillus plantarum*, *Lactobacillus fermentum*, *Lactobacillus reuteri*, *Acinetobacter radioresistens*, etc. These species were found to be highly correlated with acetic acid, lactic acid, oxidative acid, and other flavor compounds. Additionally, the top 10 differential core fungi were *Unclassified*, *Aspergillus flavus*, *Meyerozyma caribbica*, *Candida mucifera*, *Aspergillus penicillioides*, *Pichia myanmarensis*, which were strongly related to citric acid, Benzeneethanol, Hexanoic acid, Furfural, Octanoic acid, Ala, and other flavor compounds.

**Figure 10:**
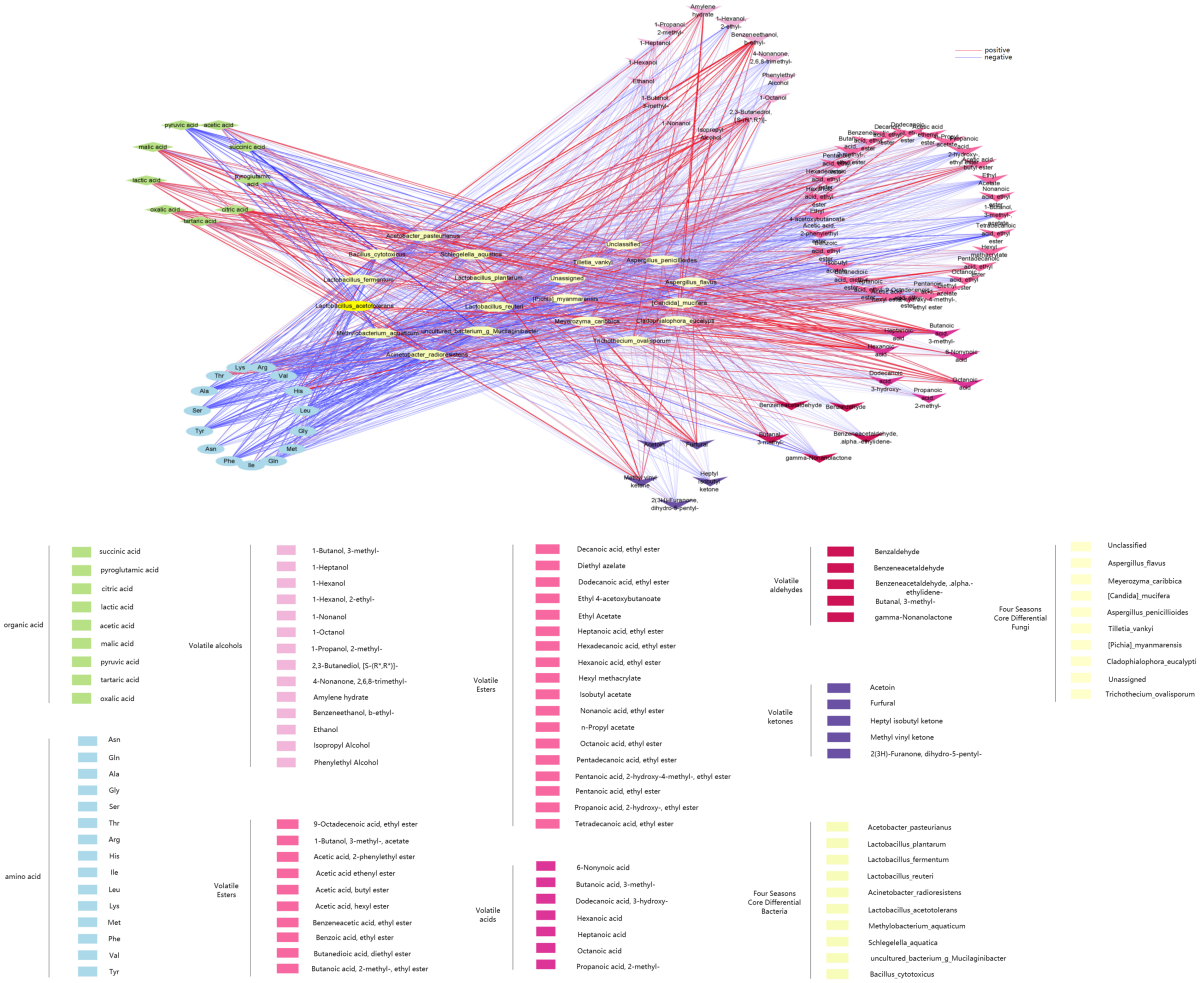
Correlation analysis chart between core microorganisms and flavor substances.

Figure 10 shows the linear analysis graph of the correlation between differential core microorganisms and flavor compounds in ZAV using Cytoscape. Combined with the correlation analysis table between differential core microorganisms and flavor compounds in Supplementary Tables S1, S2, it can be observed that the differential core microorganisms in the fermentation process of ZAV are negatively correlated with most amino acids and positively correlated with most organic acids and volatile acid compounds. Among them, *Lactobacillus acetotolerans*, *Lactobacillus plantarum*, *Lactobacillus reuteri*, and *Lactobacillus fermentum* in the differential core bacteria show a strong positive correlation with acetic acid and lactic acid. *Lactobacillus plantarum* shows a strong positive correlation with Acetoin, Hexadecanoic acid ethyl ester, and others, while showing a strong negative correlation with 9-Octadecenoic acid ethyl ester, Amylene hydrate, and others. *Acetobacter pasteurianus* shows a strong positive correlation with acetic acid, lactic acid, Asn, Hexadecanoic acid ethyl ester, and a strong negative correlation with Isopropyl Alcohol, Pentadecanoic acid ethyl ester. *Bacillus cytotoxicus* shows a strong positive correlation with lactic acid and tartaric acid, and a strong negative correlation with Benzeneacetic acid ethyl ester. Through these correlation analyses, it can be found that these *lactic acid bacteria* play a more prominent role in flavor compounds compared to other core microorganisms. In addition, *acetic acid bacteria* and *Bacillus* also make significant contributions to the AAF process.

In addition to the differential core brewing strains that significantly contribute to flavor mentioned above, there are also other microorganisms closely related to core flavor compounds. *Methylobacterium aquaticum* shows a strong positive correlation with 1-Hexanol and 1-Octanol, and a strong negative correlation with Benzeneacetic acid, ethyl ester. *Schlegelella aquatica* shows a strong positive correlation with 4-Methylcyclohexaneacetic acid, 6-Nonynoic acid, Dodecanoic acid, ethyl ester, and a strong negative correlation with 1-Octanol, Benzeneacetaldehyde, Diethyl azelate, Pentanoic acid, ethyl ester, and others. Among the differential core fungi, *Candida mucifera* shows a strong negative correlation with Benzaldehyde. *Pichia myanmarensis* shows a strong positive correlation with 1-Heptanol and pyroglutamic acid, and a strong negative correlation with Dodecanoic acid, 3-hydroxy- and Isobutyl acetate. *Aspergillus flavus* shows a strong positive correlation with 1-Heptanol. These core brewing microorganisms (*Methylobacterium aquaticum*, *Schlegelella aquatica*, *Candida mucifera*, etc.) do not directly metabolize to produce flavor compounds, but coordinate the relationship between flavor producing microorganisms such as lactic acid bacteria, spores, and acetic acid bacteria, thereby improving the synthesis of flavor compounds (Wu et al., 2014).

### 3.5 Analysis of the influence of environmental factors on differential core microorganisms of ZAV in different seasons

**Figure 11:**
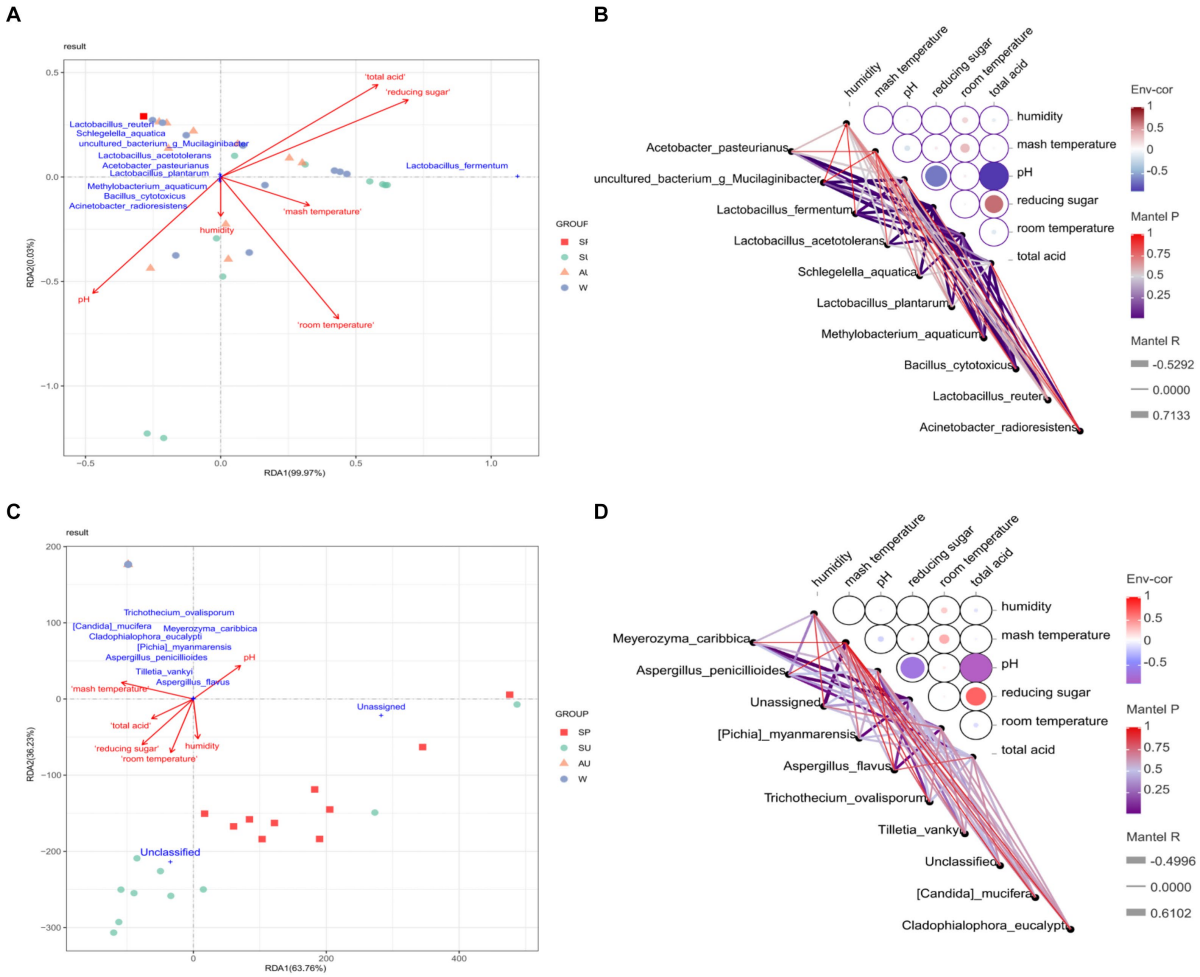
Correlation analysis of four seasons environmental factors and differential core microorganisms. **(A)** RDA analysis of the correlation between environmental factors and different core bacteria in different seasons. **(B)** Network heatmap analysis of the correlation between environmental factors and different core bacteria in different seasons. **(C)** RDA analysis of the correlation between environmental factors and different core fungi in different seasons. **(D)** Network heatmap analysis of the correlation between environmental factors and different core Fungi in different seasons.

This study conducted a redundancy analysis to analyze the correlation between environmental factors in different seasons and differential core microorganisms, and identified environmental factors highly correlated with differential core microorganisms in different seasons (Figure 11). The study found that environmental factors in different seasons significantly affect the differential core microorganisms in the acetic acid fermentation process. Total acidity, pH, reducing sugar, and room temperature showed strong correlations with differential core bacteria. *Acetobacter pasteurianus* showed a strong negative correlation with pH, while *Acetobacter pasteurianus* and *Lactobacillus acetotolerans* showed a strong positive correlation with vinegar *pei* temperature. Therefore, the amount of flavor compounds produced by *Acetobacter* and *Lactobacillus* can be controlled by adjusting the room temperature, pH, and total acidity during the acetic acid fermentation process. Furthermore, *Lachnoclostridium pacaense* was found to have a strong correlation with acetoin and ethyl decanoate. Through linear analysis of the correlation between different core microorganisms and flavor compounds in ZAV using Cytoscape, it was found that these lactic acid bacteria play a more prominent role in flavor compounds compared to other core microorganisms. These lactic acid bacteria can not only convert sugars in raw materials into lactic acid, but also produce nonvolatile organic acids such as palmitic acid, thereby enriching the flavor of vinegar. In addition, *acetic acid bacteria* and *Bacillus* also make significant contributions to the AAF process. These core vinegar fermenting bacteria (*Lactobacillus acetotolerans*, *Lactobacillus plantarum*, *Lactobacillus reuteri*, and *Lactobacillus fermentum*, *Acetobacter pasteurianus*, *Bacillus cytotoxicus*) can increase the content of ethanol and lactic acid in the fermentation system through interactions, producing volatile aroma components such as ethyl acetate with fruit aroma and isoamyl acetate with banana aroma (Román-Camacho et al., 2020). Only the presence of dominant microorganisms or flavor-promoting microorganisms cannot effectively produce flavor, and other microorganisms that do not produce flavor compounds are needed to coordinate the relationship between these flavor producing microorganisms, thereby improving the synthesis of flavor compounds (Wu et al., 2014). For example, *Pichia membranaefaciens* and *Bacillus amyloliquefaciens* are not efficient producers of flavor compounds, but they alleviate the competition between flavor producers (*Saccharomyces cerevisiae*, *I. orientalis*, and *Bacillus licheniformis*), ultimately affecting the growth and flavor production of the producers. Therefore, these core brewing strains also require special attention.

To cope with environmental changes, some microbial groups with broad physiological functions become active to adapt to new environmental conditions (Hawkes and Keitt, 2015). *Lactobacillus acetotolerans* and *Schlegelella aquatica* showed a strong positive correlation with temperature of vinegar *pei*, while *Methylobacterium aquaticum* showed a strong negative correlation with temperature. *Candida mucifera* and *Aspergillus penicillioides* also showed a strong positive correlation with the temperature of vinegar *pei*. Temperature resistance and acid resistance are one of the regulatory mechanisms for the metabolism of acetic acid and lactic acid in the fermentation process of *Acetobacter* and *Lactobacillus* (Wu Q. et al., 2021; Wu et al., 2021a,b). During the acetic acid fermentation process, the amount of flavor compounds produced by *Acetobacillus* and *Lactobacillus* can be controlled by adjusting temperature, pH, and total acidity. At the same time, *Acetobacter pasteurianus* and *Lactobacillus* showed the highest growth and metabolic activity of acetic acid and lactic acid at 30°C and 40°C, respectively. Vinegar fermentation is beneficial for the growth of *Acetobacter* and the expression of genes related to acetic acid production, but it has a negative impact on the growth of *Lactobacillus* and the expression of lactate dehydrogenase (Zheng et al., 2018). Therefore, in industrial production, synthetic biology can be used to selectively regulate and modify key metabolic pathways for acid production and acid resistance in cells, thereby solving the problem of feedback inhibition caused by the continuous accumulation of acetic acid, which leads to a decrease in cell metabolic activity (Pothimon et al., 2020; Qin et al., 2020).

The environmental humidity during the vinegar fermentation process varied significantly across different seasons (Figure 2C). The humidity levels in spring and winter were noticeably lower compared to those in summer and autumn. This could be attributed to the higher indoor temperatures during summer and autumn compared to autumn and winter (Jiang et al., 2019). Additionally, the environmental humidity during vinegar fermentation is influenced by seasonal variations, temperature, and the metabolic activity of microorganisms within the vinegar *pei* (Wang et al., 2016). Therefore, it can be inferred that the proliferation and metabolism of microorganisms in vinegar sludge will generate a large amount of heat, accelerating the evaporation of water in vinegar sludge. In Figure 11, a strong positive correlation was observed between *Lactobacillus fermentum* and humidity. This suggests that *Lactobacillus fermentum* may be more active under conditions of higher humidity. These findings demonstrate the significant influence of humidity and temperature on the growth and metabolic activity of these microorganisms, further highlighting the regulatory role of environmental factors on the microbial community in the fermentation process (Figure 11).

Reducing sugars, as important components in the vinegar fermentation process, play a crucial role. They serve as raw materials for yeast fermentation, providing the main carbon source and energy for microorganisms, and serve as precursors for the formation of vinegar flavor. The research findings show a positive correlation between *Pichia myanmarensis* and Unclassified with reducing sugars (Figure 11). Reducing sugar is a raw material for yeast fermentation, providing the main carbon source and energy for microorganisms, and also a precursor for the formation of vinegar flavor. With the development of yeast metabolic engineering and synthetic biology techniques, the utilization of yeast for organic acid production has gained increasing attention from researchers worldwide. Therefore, in industrial production, controlling the fermentation conditions of selected yeast strains through the manipulation of culture media or targeted regulation and modification of key metabolic pathways using synthetic biology can convert substrates into organic acids. This approach aims to address the challenges of low concentration, multiple by-products, and low fermentation efficiency associated with yeast-based organic acid production (Wei et al., 2023).

## 4 Discussion

Environmental factors’ seasonal shifts notably shape the microbe ecosystem and taste of fermented food by influencing environmental conditions (Zhou et al., 2020; Fang et al., 2021; Ma et al., 2022). Nevertheless, how these environmental fluctuations affect traditional ZAV’s microbes and flavor compounds is less well known. Our study delves into yearly changes in environmental elements, microbial development, and flavor compound makeup to fill this information gap. This research offers helpful seasonal perspectives on key ZAV microbes and flavor compound development.

Investigating seasonality’s impact on ZAV tasted, our study examined the aroma compound composition of vinegar *pei* across distinct seasons: concentration of acetic acid peaked in spring and augmented post-fermentation; the presence of non-volatile organic acids like lactic and succinic acids adjusts the sourness by buffering hydrogen ions to mitigate the harshness of acetic acid (Gao et al., 2021). It is worth noting that besides summer, the content of succinic acid is also high in autumn, which adds more flavor to vinegar in autumn. Succinic acid and pyroglutamic acid levels are notable in autumn, mirroring prior studies noting vinegar yields were robust in spring and winter, dominated by elevated acetic acid contents. Hence, spring/winter ZAV is perceived as sharper due to reduced sugars and amino acids. Volatile aroma compounds accumulate in autumn’s ester compounds and other flavorants contributing to its fuller flavor (Yu et al., 2012). Vinegar includes various amino acids with unique tastes—aspartic acid and glutamic acid for sourness, glycine and alanine for sweetness, methionine and arginine for bitterness, enhancing overall flavor quality (Wu Q. et al., 2021; Wu et al., 2021a,b). By conducting TAV analysis, we discerned that ZAV harvested in autumn possesses the sweetest taste, winter’s is bitter, and summer’s has distinct qualities deriving from, notably, the lowest total content of alcohols, aldehydes, ketones (Lu et al., 2018)—the result of summer’s hot weather causing a growth surge in environmental yeasts, competitive fermentations between them and beneficial microbes, potentially hampering beneficial microbe proliferation, disrupting fermentation progression and eventually reducing final vinegar’s volatiles.

Elevated dominance by *Lactobacillus* and *Acetobacillus* across all four seasons was observed during AAF via microbe community analysis. These species are responsible for major phenolic acids—acetic and lactic acids—through fermentation metabolism, also fostering ester synthesis, thereby intensifying vinegar’s unique acidity and aroma profiles (Huang et al., 2021). Interestingly, other communities displayed notable seasonality. Notably, *Acetobacter* and *Sphingomonas* topped the charts in summer, *Pseudomonas aeruginosa* and *Pseudomonas* dominated in autumn, while dominating *yeasts* were brewery and *Streptomyces genera* along with *Aspergillus* and *unassigned*, echoing prior studies (Figures 7B, 8B). It is believed that environmental factors such as temperature, acidity, and oxygen content, coupled with complex microbial interactions, mediate variations in metabolic activity leading to distinct seasonal nuanced flavors in vinegar production (Wu Q. et al., 2021; Wu et al., 2021a,b).

In traditional Chinese cereal vinegar production, a diverse microbial ecosystem is formed through fermentation involving several microbes. Research has pinpointed the essential microbiota in AAF, dominated by Lactobacillus and Acetobacter (75% + dominance) and rarer species like *Komagataribacter*, *Erwinia*, and *Methylobacterium* (Peng et al., 2021). These works primarily analyze vinegar *pei* during fixed periods, lacking seasonal environmental perspectives. Thus, these findings could provide insight into analyzing seasonally varying core microorganisms. This research aimed at identifying the primary 10 bacterial and 10 fungal core species related to flavor compounds in distinct seasons. *Acetobacter pasteurianus*, *Lactobacillus plantarum*, *Lactobacillus fermentum* amongst the bacteria, and *Aspergillus flavus*, *Meyerozyma caribbica*, *Candida mucifera* among the fungi were part of this selection. This discovery paves the way for controlled accounting of AAF microorganism population and subsequently improving the flavor quality of ZAV.

Mastering the ecological aspects of fermentation microflora allows us to precisely manage the flavor profile during vinegar creation. In this noteworthy research, we investigated the interplay between seasonal environmental elements and vital microorganisms. Notably, room temperature, overall acidity, pH level, reducing sugars, and relative humidity play a pivotal role in shaping the intricate microbial ecosystem. With the advent of sophisticated metabolic engineering and synthetic biology techniques, we are now equipped to manipulate the fermentation parameters of these pivotal core strains in commercial scale productions. Consequently, by employing culture manipulation strategies or synthetic biology methodologies, focusing on and refining crucial metabolic activities could lead to an accurate management of environmental conditions and microbial populations, enabling effective and immediate control over the flavor quality in our fermentative creations.

## 5 Conclusion

The research deeply examined the seasonal changes in Zhenjiang balsamic vinegar’s microflora and flavor compounds throughout the acetic acid fermentation (AAF) period. Autumn-brewed vinegar registered the optimal taste and sweetness due to its high levels of flavor substances. Spring showed the highest acetic acid, winter the most concentrated lactic acid, and fall the peak succinic and pyroglutamic acid. The flavor and sweetness in autumn were amplified by amino acids, whereas bitterness was notably enhanced in winter. Phylogenetic studies unveiled the dominance of *Lactobacillus* and *Acetobacter genera* alongside *Saccharomyces*, *Alternaria*, *Aspergillus*, and *Unassigned* fungi. At species-level, *Lactobacillus foodborne*, *Lactobacillus acidophilus*, and *Acetobacter pasteurella* stood out as the primary bacteria. Other microorganisms like *Lactobacillus fermentum*, *Aspergillus broom*, Rhizopus cryptorchidism, and *Pichia pastoris* also contributed to the AAF process. Differential core bacteria such as *Lactobacillus acetotolerans*, *Lactobacillus plantarum*, *Lactobacillus reuteri*, *Lactobacillus fermentum*, and *Acetobacter pasteurianus* were crucial in shaping ZAV flavor substances. The correlation analysis underscored how environmental variables like temperature, total acidity, pH, reducing sugar content, and humidity significantly affected ZAV core microbial composition and flavor profile. Synthetic biology may refine the AAF process by manipulating these key metabolic pathways of microorganisms governed by tightly regulated environmental conditions. Such strategy paves the way for contemporary ZAV manufacturing.

## Data availability statement

The datasets presented in this study can be found in online repositories. The names of the repository/repositories and accession number(s) can be found in the article/Supplementary material.

## Author contributions

XY: Writing – review & editing, Software, Writing – original draft, Visualization. YY: Writing – review & editing, Supervision, Resources, Project administration, Funding acquisition. JL: Writing – original draft, Software. YZ: Writing – review & editing, Writing – original draft, Formal analysis, Data curation. ZY: Writing – original draft, Methodology. PL: Writing – original draft, Investigation. YW: Writing – review & editing, Investigation, Formal analysis. KW: Writing – review & editing, Validation, Conceptualization.
